# Osteoprotegerin is a significant prognostic factor for overall survival in patients with primary systemic amyloidosis independent of the Mayo staging

**DOI:** 10.1038/bcj.2015.45

**Published:** 2015-06-05

**Authors:** E Kastritis, M Gavriatopoulou, M A Dimopoulos, E Eleutherakis-Papaiakovou, N Kanellias, M Roussou, C Pamboucas, S T Toumanidis, E Terpos

**Affiliations:** 1 Department of Clinical Therapeutics, National and Kapodistrian University of Athens, School of Medicine, Athens, Greece

## Abstract

Bone metabolism has not been systematically studied in primary (AL) amyloidosis. Thus we prospectively evaluated bone remodeling indices in 102 patients with newly diagnosed AL amyloidosis, 35 healthy controls, 35 newly diagnosed myeloma and 40 monoclonal gammopathy of undetermined significance patients. Bone resorption markers (C-telopeptide of type-1 collagen, N-telopeptide of type-1 collagen) and osteoclast regulators (soluble receptor activator of nuclear factor-κB ligand (sRANKL), osteoprotegerin (OPG)) were increased in AL patients compared with controls (*P*<0.01), but bone formation was unaffected. Myeloma patients had increased bone resorption and decreased bone formation compared with AL patients, while sRANKL/OPG ratio was markedly decreased in AL, due to elevated OPG in AL (*P*<0.001). OPG correlated with N-terminal pro-brain natriuretic peptide (*P*<0.001) and was higher in patients with cardiac involvement (*P*=0.028) and advanced Mayo stage (*P*=0.001). OPG levels above the upper value of healthy controls was associated with shorter survival (34 versus 91 months; *P*=0.026), while AL patients with OPG levels in the top quartile had very short survival (12 versus 58 months; *P*=0.024). In Mayo stage 1 disease, OPG identified patients with poor survival (12 versus >60 months; *P*=0.012). We conclude that increased OPG in AL is not only a compensation to osteoclast activation but may also reflect early cardiac damage and may identify patients at increased risk of death within those with earlier Mayo stage.

## Introduction

Primary systemic (AL) amyloidosis is a rare disease characterized by deposits of monoclonal immunoglobulin light chain-derived amyloid fibrils in vital organs. Widespread deposition of amyloid fibrils causes multisystem organ dysfunction.^1^ Kidneys, heart, liver, nerves, gastrointestinal tract and soft tissues are most commonly involved.^2^ Deregulation of bone metabolism is common in plasma cell dyscrasias, including multiple myeloma (MM) and monoclonal gammopathy of undetermined significance (MGUS),^3, 4, 5^ but very little is known for bone remodeling in patients with AL amyloidosis. Bone manifestations are considered to be rare in AL patients and include mainly vertebral amyloidomas, characteristic circumscribed osteolytic lesions due to amyloid deposits and chronic arthropathies.^6, 7, 8, 9^ In the largest published series, Schonland *et al.*^10^ identified four cases among 330 AL patients with osteolytic lesions in the absence of MM. The pathophysiology of the development of these lesions seems to be the local production of amyloid fibrils by clonal plasma cells, but this has been confirmed only in a minority of the published cases with histomorphometry studies. In the above study, the authors query whether diffuse amyloid deposits may alter bone remodeling leading to bone loss.^10^ However, to date, there was no systematic study of bone metabolism in patients with AL amyloidosis.

The aim of our prospective study was to evaluate bone metabolism in newly diagnosed patients with AL amyloidosis, to compare the results with those of patients with other plasma cell neoplasms, including MM and MGUS, and explore possible correlations with disease characteristics, including survival.

## Patients and methods

### Study design

This was a prospective study for the evaluation of bone parameters in patients with AL amyloidosis and their correlation with features of the disease, such as organ involvement, Mayo stage and survival.

#### Inclusion and exclusion criteria

The inclusion criteria of the study included: (i) adult patients with previously untreated AL amyloidosis in whom diagnosis was based on consensus criteria;^11^ and (ii) patients who have given their written informed consent for blood sampling and for recording of their medical data, which is pertinent to the purposes of this study.

The exclusion criteria included: (i) patients aged <18 years; (ii) the presence of more than one lytic bone lesions in plain radiography or other features suggestive of MM, such as hypercalcemia, significant anemia unrelated to renal impairment or predominant Bence–Jones proteinuria; (iii) clinical history of other bone disease (that is, osteoporosis, osteoarthritis, Paget's disease and so on); and (iv) use of medication that could alter the normal bone turnover during the past 6 months (that is, bisphosphonates, denosumab, teriparatide and so on).

#### Study end points

The primary end point of the study was the evaluation of biochemical parameters of bone remodeling in AL patients at the time of diagnosis and their comparison with those of healthy controls, MM and MGUS patients.

Secondary end points included: (i) correlation of biochemical markers of bone remodeling with organ involvement (heart, renal, bone and so on); (ii) correlation of bone markers with monoclonal plasma cell infiltration; and (iii) correlation of bone markers with patients' overall survival (OS).

#### Patients' enrolment

The enrolment period was between January 2000 and January 2011. Patients were informed of the objectives and details of the present study before they gave their approval and signed the informed consent form. The study was conducted according to the principles defined by the Eighteenth World Medical Association Assembly (Declaration of Helsinki, 1964) and all its future amendments. The study protocol was designed and executed according to the guidelines and regulations pertaining to studies in Greece as well as the Good Clinical Practice Guidelines as defined by the International Conference of Harmonization. The study was approved by the local ethical committee.

#### Enrolment of the control groups

In this study, bone remodeling markers were also measured in 35 age- and gender-matched healthy controls (15 males/20 females, median age 65 years), 40 patients with MGUS (17 males/23 females, median age 66 years) and 35 newly diagnosed, symptomatic patients with MM (14 males/21 females, median age 65 years) before the administration of any kind of therapy. MGUS and MM patients had similar age and gender with the AL patients and were diagnosed during the same recruitment period. The medical history of healthy controls and MGUS patients was recorded in order to assure that they had no history of bone disorder (that is, osteoporosis, osteoarthritis) and did not receive any drug that could alter bone metabolism during the past 6 months.

#### Data recording and quality assurance

Data were collected from the medical files of the patients. Clinical study monitors performed source data verifications and ensured the accuracy of these data. Among other data, we recorded treatment data, treatment outcome according to consensus criteria^12^ and patients' OS.

#### Statistical analysis

Mann–Whitney and Kruskal–Wallis tests were used for the comparison of continuous variables among different groups. Chi-square test was used for categorical variables, using Fisher's exact test when appropriate. Spearman's nonparametric correlation coefficient and linear regression were used for the identification of significant correlations between continuous variables. Logarithmic transformation was also used for heavily skewed variables. Survival was calculated from the day of treatment initiation until the date of death or last follow-up. Patients who were lost to follow-up were censored at the date of last contact. Patients who died before any organ or hematological response could be assessed and were rated as having both hematological and organ progression. Time-to-event curves were plotted with the method of Kaplan and Meier, and comparisons were made using the log-rank test. Cox proportional hazards models were used for time-to-event multivariate analysis. All analyses were performed using the SPSS 20 software (IBM SPSS statistics for Windows, Version 20.0, IBM Corp, Armonk, NY, USA).

### Measurement of bone remodeling indices

After vein puncture, serum was separated within 4 h and stored at −80 °C for all patients and controls. An enzyme-linked immunosorbent assay was used for the detection of the following serum indices: (i) osteoclast regulators: soluble receptor activator of nuclear factor-κ B ligand (sRANKL) and osteoprotegerin (OPG) (Biomedica, Wien, Austria); (ii) bone resorption markers: C-telopeptide of type-1 collagen (CTX; serum CrossLaps, Immunodiagnostic Systems Nordic a/s, Herlev, Denmark), N-telopeptide of type-1 collagen (NTX; serum Osteomark, Alere Inc., Scarborough, ME, USA) and tartrate-resistant acid phosphatase type-5b (TRACP-5b; BoneTRAP, Immunodiagnostic Systems Ltd., Boldon, Tyne and Wear, UK); and (iii) bone formation markers: bone-alkaline phosphatase (bALP; Metra BAP, Quidel Co., San Diego, CA, USA) and osteocalcin (OC; N/MID Osteocalcin, Immunodiagnostic Systems Nordic a/s).

## Results

### Patients and controls

In total, 102 patients with previously untreated AL amyloidosis participated in this study. Patients' characteristics are depicted in Table 1. The median age was 65 years and 43% were males. Heart involvement was established in 60% of the patients and renal involvement in 73%, while 67% of patients had two or more organs involved. At the time of enrolment, 16% had a baseline serum creatinine >2 mg/dl and 19% estimated glomerular filtration rate (eGFR) (calculated with the MDRD formula) <30 ml/min/1.73 m^2^. None of the AL patients had lytic bone lesions in plain radiography.

Most patients received primary therapy with novel agents (bortezomib-based in 25% and lenalidomide-based in 40%), while 30% received therapy with melphalan and dexamethasone and 5% with high-dose dexamethasone-based regimens (mainly VAD).

Regarding MGUS patients, 26/40 (65%) had immunoglobulin G (IgG) monoclonal protein, 11 (27.5%) IgA and 3 (7.5%) light chain only M-component; 27 (67.5%) had kappa and 13 (32.5%) lambda light chain. Out of the 35 studied myeloma patients, 16 (46%) had IgG MM, 11 (31%) IgA and 8 (23%) light chain only MM. International Staging System (ISS)-1, ISS-2 and ISS-3 MM had 11 (32%), 12 (34%) and 12 (34%) patients, respectively. In plain X-rays, 24 (68.5%) patients had osteolytic lesions, while 7 (20%) had a pathological fracture at the time of diagnosis.

### Markers of bone remodeling in all the patients and control groups

**Figure 1:**
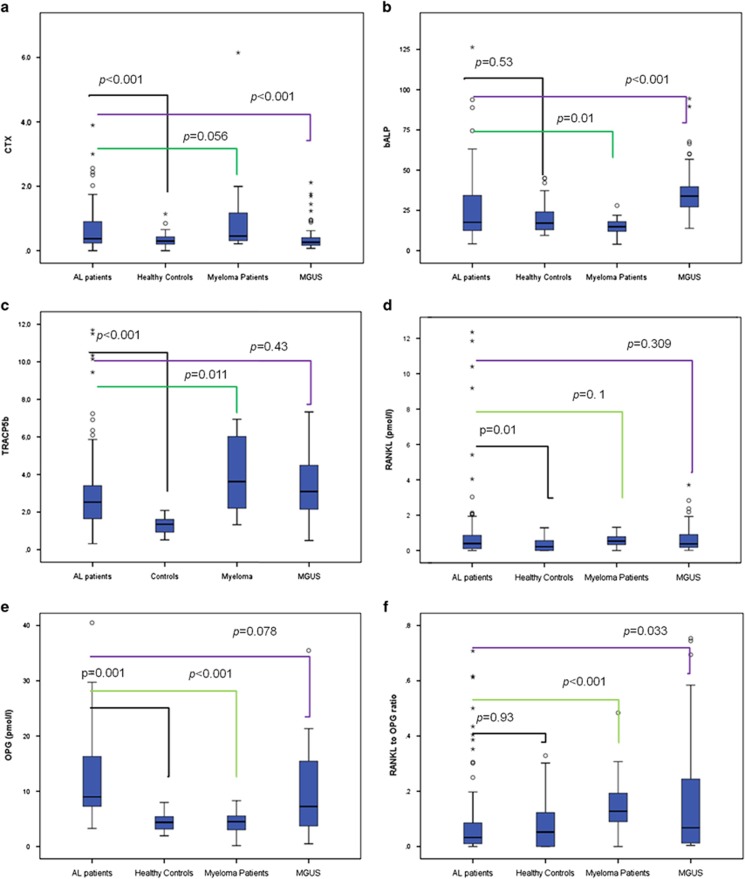
Markers of bone remodeling in AL patients and other study groups: CTX in ng/ml (**a**), bALP in U/l (**b**), TRACP-5b in U/l (**c**), sRANKL in pmol/l (**d**), OPG in pmol/l (**e**) and sRANKL/OPG ratio (**f**).

Bone resorption, as assessed by both bone degradation products (CTX and NTX) and TRACP-5b values, was increased in patients with AL amyloidosis versus healthy controls (*P*<0.001 for all comparisons; Table 2; Figures 1a and c). On the other hand, there was no difference regarding markers of bone formation (bALP and OC) between AL patients and healthy controls (Table 2; Figure 1b). Osteoclast regulator's levels (sRANKL, OPG) were all increased compared with controls (*P*<0.01 for all comparisons; Table 2). However, the concomitant increase of both sRANKL and OPG in AL patients resulted in a similar sRANKL/OPG ratio with that of healthy controls (Table 2; Figures 1d–f).

MM patients had increased bone resorption and decreased bone formation markers compared with AL patients, while sRANKL/OPG ratio was markedly decreased in AL versus MM, due to high levels of OPG in AL patients compared with MM patients (median was approximately twofold higher in AL; *P*<0.001). Compared with MGUS, AL patients had decreased sRANKL/OPG ratio due to increased circulating OPG.

**Figure 2:**
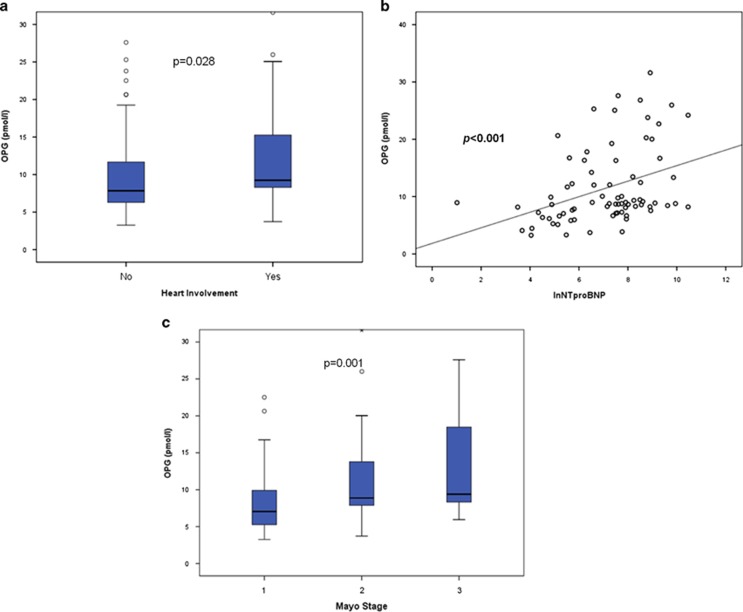
Circulating OPG in AL patients: patients with heart involvement had higher OPG levels (**a**). OPG correlated with NT-proBNP (**b**) and Mayo disease stage (**c**).

In AL patients, OPG levels correlated with N-terminal pro-brain natriuretic peptide (NT-proBNP) levels (*r*=0.41, *P*<0.001; Figure 2a) and were slightly higher in patients with cardiac involvement (*P*=0.028; Figure 2b). OPG levels also correlated with Mayo stage (*P*-ANOVA=0.001; Figure 2c). Although renal involvement did not affect other bone markers, in AL patients with eGFR<30 ml/min/1.73 m^2^ or eGFR< 60 ml/min/1.73 m^2^, CTX and NTX were higher than in patients eGFR above these cutoffs (*P*<0.001 and *P*=0.016, respectively). OPG levels inversely correlated with eGFR (*r*=−0.323, *P*=0.002). The degree of bone marrow plasma cell infiltration was not correlated to RANKL or OPG levels or their ratio. Neither the involvement of other organs nor the number of involved organs did not appear to influence bone remodeling in any way. There was no correlation of any of the bone indices with the levels of involved free light chains. No other significant correlations were observed among markers of bone remodeling and disease characteristics in AL patients.

### Bone markers and survival

**Figure 3:**
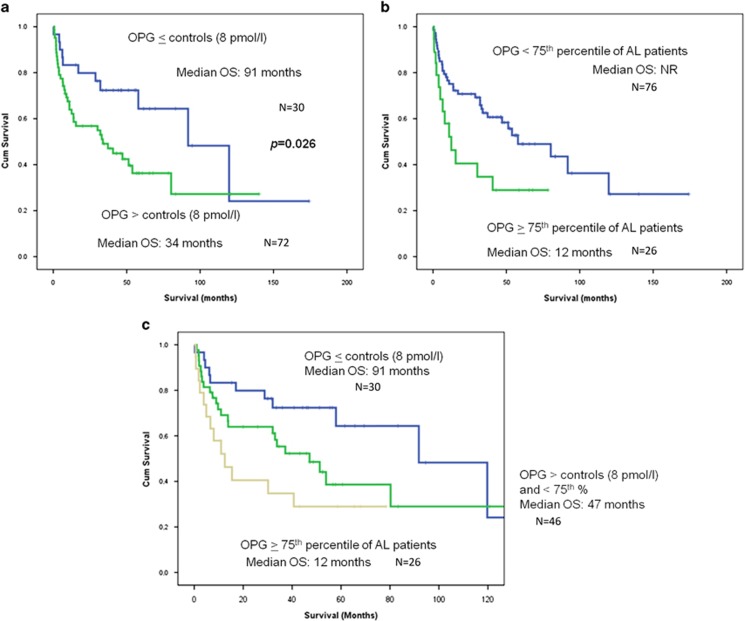
Circulating OPG and survival in AL patients: Patients with OPG levels above the upper limit of healthy controls (8 pmol/l) (*N*=72) had lower survival (34 months) compared with all others (*N*=72) (91 months) (**a**). AL patients with OPG of the higher quartile (16.3 pmol/l) (*N*=26) had a very poor survival of 12 months (**b**). Patients with OPG values between 8 and 16.3 pmol/l (*N*=46) had an overall survival between the previous groups of AL patients: 47 months (**c**).

After a median follow-up of 55 months, 51 (50%) patients have died, and the median survival of the cohort was 54 months. We found that only OPG, out of all studied markers of bone metabolism, correlated with survival in AL patients. More specifically, patients with OPG levels above the upper value of healthy controls (8 pmol/l) had a shorter survival (34 versus 91 months; *P*=0.026; Figure 3a). Furthermore, AL patients with OPG levels in the top quartile (16.3 pmol/l) had very poor survival (12 versus 58 months; *P*=0.024; Figure 3b). If we use the above two OPG values (8 and 16.3 pmol/l), we could identify three groups of AL patients with different survival: those who had an OPG value of ⩽8 pmol/l, those with OPG between 8 and 16.3 pmol/l, and those with OPG⩾16.3 pmol/l with median OS of 91, 47 and 12 months, respectively (Figure 3c).

**Figure 4:**
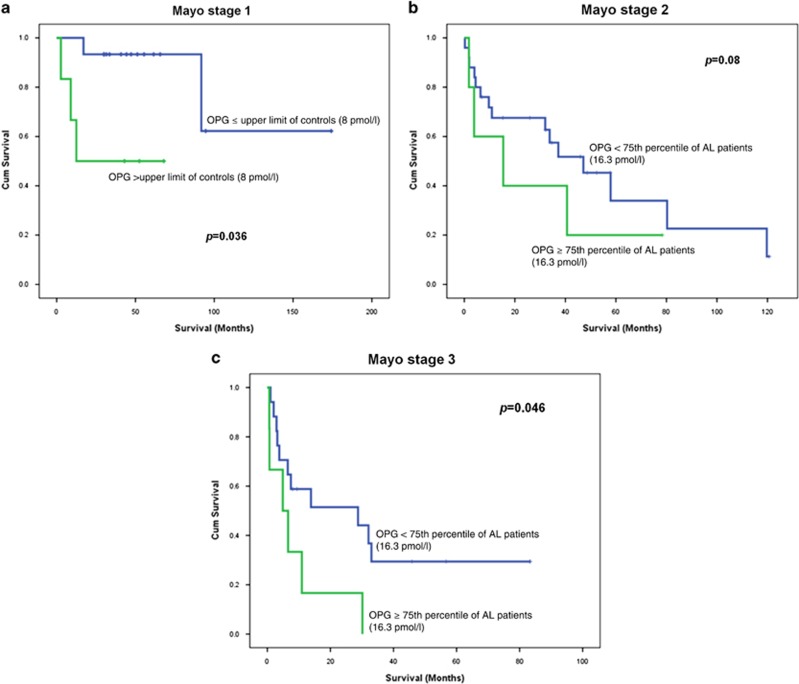
OPG could identify groups of patients with shorter survival among the three Mayo stages.

Because OPG levels correlated with NT-proBNP levels, the strongest predictor of survival in patients with AL, we performed a multivariate analysis, in which cardiac biomarkers outperformed OPG, probably because of their correlation. However, OPG⩾16.3 pmol/l (75th percentile of AL patients) remained a poor prognostic factor independent of Mayo stage (Table 3). In addition, patients with Mayo stage 1 disease and OPG levels >8 pmol/l had a very short survival (12 versus >60 months, *P*=0.012; Figure 4a), while OPG level >16.3 pmol/l (75th quartile) further dissected Mayo stages 2 and 3 AL patients (Figures 4b and c). Thus OPG had prognostic significance, which was additional to traditional prognostic markers.

## Discussion

Systemic light chain (AL) amyloidosis presents unique challenges both in understanding the biology and for the therapy of the affected individuals. The presence of a plasma cell clone in the unique microenvironment of the bone marrow causes multiple organ dysfunction through the production of the amyloidogenic light chains, while within the bone marrow microenvironment the plasma cells interact with either cellular or non-cellular components of the bone, but no data exist about this interaction. The aim of this study was to evaluate, for the first time in the literature, the bone metabolism of patients with AL amyloidosis. First, we found that bone resorption is increased compared with healthy controls. The most accurate markers of bone collagen degradation, CTX and NTX,^13^ were both increased in AL patients, and moreover, TRACP-5b, which is produced only by activated osteoclasts,^14^ was also elevated in AL patients compared with healthy individuals. Thus, increased osteoclast activity is present in AL patients, although we found no lytic lesions in the skeletal radiography. This is in accordance with a report of the Heidelberg group, where the authors found lytic lesions in only 1.2% of 330 patients with AL amyloidosis.^10^ To further investigate the lack of osteolytic lesions in AL patients despite the increased osteoclast activity, we compared the results of AL patients with those of patients with newly diagnosed, symptomatic MM who were diagnosed at the same time in our center. As expected, MM patients had higher values of all markers of bone resorption and reduced levels of markers of bone formation compared with AL patients.^15, 16, 17^ MM patients had very low bALP and OC levels compared with AL, MGUS and healthy controls. On the contrary, in AL amyloidosis, circulating levels of bone formation markers (bALP and OC) were similar to those of healthy individuals. Although sRANKL, the most potent osteoclast activator, was elevated in AL patients, this elevation was balanced by a remarkable increase of the decoy receptor of RANKL, OPG. This increase of OPG in AL led to a sRANKL/OPG ratio similar to that of healthy individuals but lower than that of MM and MGUS patients. The ratio of sRANKL/OPG is elevated in MM and correlates with the extent of bone disease^18^ but remains within normal range in other plasma cell disorders, including MGUS^4^ and Waldenström macroglobulinaemia^5^ that do not present osteolytic lesions. In MM, plasma cells reduce the levels of OPG in the bone marrow microenvironment.^19^ Possibly, the low number of malignant plasma cells that are present in AL amyloidosis cannot inhibit osteoblast function and cannot reduce the OPG level in the AL microenvironment, as in MM. On the contrary, OPG was overproduced in our AL patients and fully compensated the increase of osteoclast activity and may partly explain the lack of osteolytic lesions in our patients.

The most important finding of our study was the correlation of the aforementioned elevated OPG levels with AL patients' survival: OPG levels, either >8 pmol/l (the upper limit of healthy controls) or >16.3 pmol/l (the 75th quartile of AL patients), were able to identify three groups of patients with different median OS: 12, 47, and 91 months, respectively (Figure 3c). The correlation of OPG with survival cannot be only explained by its overproduction by osteoblasts or other marrow stromal cells to override the osteoclast activation. In our study, no other bone marker correlated with OS; in contrast to MM where several markers of bone remodeling are associated with OS, that is, CTX,^20^ NTX,^16^ sRANKL/OPG ratio^18^ or macrophage inflammatory protein one alpha.^21^ Cardiac involvement is considered as the most adverse prognostic factor in patients with AL,^23^ and the cardiac biomarkers NT-proBNP and troponins are the most important prognostic factors for OS.^22^ During the past decade, OPG has been implicated in the pathogenesis of heart failure of different etiologies. Levels of circulating OPG have been associated with the incidence of death, independently of conventional cardiovascular risk factors, in patients with heart failure,^24^ even in those with preserved ejection fraction.^25^ Furthermore, OPG independently predicted deterioration of heart failure that required hospitalization, mainly in older patients with advanced chronic systolic heart failure of ischemic etiology.^26^ OPG has also provided independent prognostic information across the range of acute coronary syndromes^27, 28, 29^ and acute ischemic stroke.^30^ This is the first study, to date, which showed that OPG also provides important prognostic information in patients with AL amyloidosis. In our study, 60% of the patients had heart involvement and these patients had elevated OPG levels compared with the others (*P*=0.026), while patients with Mayo stage 3 had higher OPG levels compared with patients with stage 1 and stage 2 disease. However, OPG was a significant prognostic factor independently of the Mayo stage. More specifically, OPG could identify a subgroup of patients with very poor prognosis (median OS of 12 months) even within the Mayo stage 1, where patients have low NT-proBNP and low troponin levels. One possible explanation for the prognostic significance of OPG in AL amyloidosis is that OPG is produced and secreted by the failing myocardium,^31^ possibly earlier than NT-proBNP and troponin. Another hypothesis is that the association of circulating OPG with prognosis reflects the generalized inflammation and the vascular disease of AL, which further contributes to heart dysfunction due to the fact that the heart is working against an increasingly atherosclerotic and stiff vascular tree. But these hypotheses have to be proven in experimental models.

In conclusion, our study supports the presence of increased bone resorption and osteoclast activation that is fully compensated and thus osteolytic lesions are rare in AL amyloidosis. The increased levels of OPG in AL amyloidosis correlate with prognosis independently of the Mayo staging system, and this suggests that OPG levels not only represent a balancing effect to the increased osteoclast function but also reflect an early heart failure phenomenon and/or a generalized vascular defect in AL amyloidosis. Our results highlight the role of OPG in the biology of AL amyloidosis and support the broader use of this marker for the confirmation of its prognostic significance.

## Figures and Tables

**Table 1:** Characteristics of patients with AL amyloidosis

No. of patients	102
Age, years, median (range)	65 (39–80)
Gender (M/F), *n*	44/58
	
*Organ involvement*	
Cardiac	60%
Renal	73%
Liver	11%
PNS/ANS	40%
Soft tissue	28%
	
No. of involved organs, median (range)	2 (1–4)
proBNP, pg/ml, median (range)	1954 (80–75000)
Mayo stage-1	26%
Mayo stage-2	41%
Mayo stage-3	33%
Proteinuria, g/day, median (range)	3.35 (0.5–29)
eGFR, ml/min/1.73 m^2^, median (range)	72 (8–210)
eGFR <30 ml/min/1.73 m^2^	19%
Serum albumin, g/dl, median (range)	3.2 (2.5–4.0)
Bone marrow plasma cells, %, median (range)	12 (1-30)
Involved FLC, mg/l, median (range)	181 (11–1510)
Beta2-microglobulin, mg/l, median (range)	1.8 (1.5–4.5)

Abbeviations: AL, primary amyloidosis; ANS, autonomous nervous system; F, female; FLC, free light chains; eGFR, estimated glomerular filtration rate; M, male; PNS, periphery nervous system; proBNP, pro-brain natriuretic peptide.

**Table 2:** Baseline values of bone metabolism parameters in all the study groups; mean±s.d. (median)

*Variable*	*AL (*N=*102)*	*Healthy controls (*N=*35)*	*Multiple myeloma (*N=*35)*	*MGUS (*N=*40)*
CTX (ng/ml)	0.65±0.67	0.34±0.23	0.87±1.05	0.37±0.37
	(0.37)	(0.3)	(0.453)	(0.26)
NTX (nmol BCE)	18.2±13.3	9.7±3.43	15.76±7.71	8.057±4.31
	(13.2)	(9.77)	(15.07)	(7.037)
TRACP-5b (U/l)	3.29±2.66	1.28±0.34	4±1.83	3.25±1.65
	(2.53)	(1.35)	(3.62)	(3.09)
bALP (U/l)	25.1±19.8	19.8±9.65	14.7±5.1	37.2±18.36
	(17.6)	(17.1)	(14.85)	(33.9)
OC (ng/ml)	16.8±16.02	18.48±10.08	13.08±5.1	10.59±8.94
	(12.3)	(15.1)	(13.5)	(7.29)
OPG (pmol/l)	12.1±7.35	4.4±1.55	4.34±1.74	10.4±9.3
	(8.98)	(4.37)	(4.49)	(7.24)
RANKL (pmol/l)	1.02±2.19	0.32±0.36	0.59±0.31	0.75±0.84
	(0.395)	(0.219)	(0.53)	(0.374)
RANKL/OPG ratio	0.085±0.141	0.082±0.096	0.24±0.51	0.163±0.219
	(0.0325)	(0.052)	(0.128)	(0.068)

Abbeviations: AL, primary amyloidosis; bALP, bone-alkaline phosphatase; CTX, C-telopeptide of type-1 collagen; MGUS, monoclonal gammopathy of undetermined significance; NTX, N-telopeptide of type-1 collagen; OC, osteocalcin; OPG, osteoprotegerin; RANKL, receptor activator of nuclear factor-κB ligand; TRACP-5b, tartrate-resistant acid phosphatase type-5b.

**Table 3:** Multivariate analysis for the significance of osteprotegerin on survival of AL patients

	HR	95% CI		P-value
OPG⩽8 pmol/l	1			
OPG>8 and <16.32	1.20	0.53	2.88	0.66
OPG⩾16.32 pmol/l	2.30	1.14	5.77	0.049
Mayo-1	1			
Mayo-2	2.67	1.06	7.68	0.029
Mayo-3	4.40	1.54	13.3	0.009
FLC⩾180 mg/l	1.89	0.948	3.64	0.071

Abbeviations: AL, primary amyloidosis; CI, confidence interval; FLC, free light chains; HR, hazard ratio; OPG, osteoprotegerin.
